# Evaluation of Antidiabetic, Antioxidant and Anti-Hyperlipidemic Effects of *Solanum indicum* Fruit Extract in Streptozotocin-Induced Diabetic Rats

**DOI:** 10.3390/cimb45020058

**Published:** 2023-01-19

**Authors:** Manoj M. Gadewar, Prashanth G K, Prabhu Chandra Mishra, Ghulam Md Ashraf, Majed N. Almashjary, Steve Harakeh, Vijay Upadhye, Abhijit Dey, Pallavi Singh, Niraj Kumar Jha, Saurabh Kumar Jha

**Affiliations:** 1 Department of Pharmacology, School of Medical and Allied Sciences, K.R. Mangalam University, Gurgaon 122103, India; 2 Department of Chemistry, Sir M. Visvesvaraya Institute of Technology, Visvesvaraya Technological University, Bengaluru 562157, India; 3 Department of Biotechnology, School of Engineering and Technology, Sharda University, Greater Noida 201310, India; 4 Department of Medical Laboratory Sciences, College of Health Sciences, and Sharjah Institute for Medical Research, University of Sharjah, Sharjah 27272, United Arab Emirates; 5 Department of Medical Laboratory Sciences, Faculty of Applied Medical Sciences, King Abdulaziz University, Jeddah 21589, Saudi Arabia; 6 Hematology Research Unit, King Fahd Medical Research Center, King Abdulaziz University, Jeddah 21589, Saudi Arabia; 7 Animal House Unit, King Fahd Medical Research Center, King Abdulaziz University, Jeddah 21589, Saudi Arabia; 8 King Fahd Medical Research Center, King Abdulaziz University, Jeddah 21589, Saudi Arabia; 9 Prophetic Medicine Application, Faculty of Medicine (FM), King Abdulaziz University, Jeddah 21589, Saudi Arabia; 10 Centre of Research for Development (CR4D), Parul University (DSIR-SIRO Recognized) PO Limda, Tal Waghodiya, Vadodara 391760, India; 11 Department of Life Sciences, Presidency University, 86/1 College Street, Kolkata 700032, India; 12 Department of Biotechnology, Graphic Era Deemed to be University, Dehradun 248002, India; 13 School of Bioengineering & Biosciences, Lovely Professional University, Phagwara 144411, India; 14 Department of Biotechnology Engineering and Food Technology, Chandigarh University, Mohali 140413, India; 15 Department of Biotechnology, School of Applied & Life Sciences (SALS), Uttaranchal University, Dehradun 248007, India

**Keywords:** *Solanum indicum*, streptozotocin, antioxidants, lipid profile, antidiabetic

## Abstract

Background: Globally, diabetes mellitus is the most common cause of premature mortality after cardiovascular diseases and tobacco chewing. It is a heterogeneous metabolic disorder characterised by the faulty metabolism of carbohydrates, fats and proteins as a result of defects in insulin secretion or resistance. It was estimated that approximately 463 million of the adult population are suffering from diabetes mellitus, which may grow up to 700 million by 2045. *Solanum indicum* is distributed all over India and all of the tropical and subtropical regions of the world. The different parts of the plant such as the roots, leaves and fruits were used traditionally in the treatment of cough, asthma and rhinitis. However, the hypoglycaemic activity of the plant is not scientifically validated. Purpose: The present study aimed to evaluate the antioxidant, antidiabetic and anti-hyperlipidaemic activity of methanolic fruit extract of *Solanum indicum* (SIE) in streptozotocin (STZ) induced diabetic rats. Method: Experimentally, type II diabetes was induced in rats by an i.p. injection of STZ at a dose of 60 mg/kg. The effect of the fruit extract was evaluated at doses of 100 and 200 mg/kg body weight in STZ-induced diabetic rats for 30 days. Result: The oral administration of fruit extract caused a significant (*p* < 0.05) reduction in the blood glucose level with a more prominent effect at 200 mg/kg. The fruit extract showed dose-dependent α-amylase and α-glycosidase inhibitory activity. It reduced the serum cholesterol and triglyceride levels remarkably in diabetic rats compared to normal. The extract showed the reduced activity of endogenous antioxidants, superoxide dismutase, glutathione peroxidase and catalase in the liver of STZ diabetic rats. Conclusion: The result confirmed that the fruit extract of *Solanum indicum* showed a dose-dependent blood glucose lowering effect and significantly reduced elevated blood cholesterol and triglycerides. It prevented oxidative stress associated with type II diabetes in STZ rats.

## 1. Introduction

Diabetes mellitus is a heterogeneous metabolic disease characterised by the faulty metabolism of carbohydrates, fats and proteins [1] as a result of defects in insulin secretion or resistance. It was estimated that approximately 463 million adults suffer from diabetes mellitus, which may grow to up to 700 million by 2045. Globally, it is the most common cause of premature mortality after cardiovascular diseases and tobacco chewing [2]. Hyperglycaemia in diabetes causes lipid peroxidation and membrane damage due to the excessive production of oxygen-derived free radicals which ameliorate the development of secondary complications such as retinopathy, neuropathy and nephropathy. Cardiovascular complications result in the deposition of fatty plaques in small-diameter blood vessels and further hardening and narrowing (atherosclerosis) that could advance to cerebral stroke, acute or chronic renal failure, loss of sensation and permanent blindness [3]. Antioxidants derived from the plant prevent the destruction of β cells by inhibiting the peroxidation chain reaction, thus lowering the oxidative stress in diabetes [4]. Given that the spectrum of currently available drugs used in the treatment of diabetes have limitations of their own; many herbal medicines have been used and recommended for the treatment of diabetes [5]; various phytoconstituents present in the medicinal plant act on different targets by various mechanisms and are responsible for producing therapeutic actions [6]. The plant *Solanum indicum* Linn (Solanaceae) is widely distributed in India and all over the tropical and subtropical regions of the world [7]. The different parts (fruits, leaves, roots) of the plant were used traditionally for the treatment of rhinitis, cough, asthma, sore throat, hiccup, sexual disorders, abdominal pain, worm infestation, fever, inflammation, insomnia, urinary complications, cardiac weakness and blood disorders, etc. Despite a growing body of evidence revealing the therapeutic potential of the crude extracts of different *Solanum* species on experimentally induced diabetes in animals [8,9,10], the antidiabetic potential of the *Solanum indicum* fruits has not been scientifically explored so far. Thus, the present study has been undertaken to evaluate the hypoglycaemic and anti-hyperlipidemic effects of the fruit extract of *Solanum indicum* in streptozotocin (STZ)-induced diabetic rats.

## 2. Materials and Methods

### 2.1. Chemicals and Reagents

STZ, 2, 2-diphenyl-1-picrylhydrazyl (DPPH), (3-(4, 5-Dimethylthiazol-2-yl)-2, 5-Diphenyltetrazolium Bromide) (MTT), porcine pancreatic α-amylase (PPA), modified eagle media and foetal bovine serum were procured from Sigma-Aldrich, Burlington, MA, USA. The Folin–Ciocalteau reagent was purchased from Himedia, Mumbai. Mouse fibroblast cell (L929) cell lines were obtained from the National Centre for Cell Sciences, Pune, India.

### 2.2. Assay Kits

The glucose, triglycerides, cholesterol, superoxide dismutase (SOD), glutathione peroxidase (GPx), catalase (CAT), alanine transaminase (ALT) and aspartate transaminase (AST) estimation kits were procured from Merck, Mumbai, India. All reagents and solvents used were of analytical grades.

### 2.3. Collection and Identification of Plant

The fruits of *Solanum indicum* were collected from the vicinity of IIT Guwahati, India. It was identified and authenticated by the Taxonomist at the Department of Botany, Guwahati University. The voucher specimen (17,783) was deposited in the Dept. of Botany, Guwahati University, Guwahati, Assam (India).

### 2.4. Extraction

The unripe fruits of *Solanum indicum* were collected during the month of March; they were then repeatedly washed with distilled water to remove soil and dirt matter and shade dried to remove excess moisture. Approximately 500 g of powder was extracted with methanol by the Soxhlet extraction method for 24 h. After 24 h, the extract was concentrated by using a rotary vacuum evaporator (Panchun Scientific Co., Kaohsiung, Taiwan). The concentrated extract was lyophilised to obtain a powder (yield 13.8% *w*/*w*) that was used for further experimentation.

### 2.5. Estimation of Total Phenolic Content

The phenolic content in the fruit extract of *Solanum indicum* was estimated as per the procedure [11]. In brief, 1 mg/mL stock solution of extract was prepared in methanol. From the stock solution, different concentrations ranging from 10–100 µg/mL (1 mL each) to this 1.25 mL 10% Folin–Ciocalteu’s reagent were added, followed by the addition of 2.5 mL of 20% sodium carbonate after 5 min, and the solutions were incubated at room temperature for 30 min.

The absorbance of the sample was measured at 765 nm; the same procedure was carried out to construct the calibration curve of gallic acid. The results were compared to the standard calibration curve of gallic acid, and the total phenolic content of the fruit extract was expressed as mg of gallic acid equivalents per gram of the extract.

### 2.6. DPPH Radical Scavenging Assay

The free radical scavenging activity of the extract was estimated as per [12] with slight modification. In brief, 1 mg/mL stock solution of extract was prepared in methanol, and dilutions were prepared to obtain different concentrations ranging from (10–100 μg/mL). A diluted solution (20 μL) of the extract was mixed with 180 μL of 0.1 mM methanolic solution of DPPH in darkness and incubated for 30 min at room temperature; the absorbance was measured at 517 nm, and the percentage radical scavenging activity was calculated using the formula,

% radical scavenging activity = Absorbance of control − Absorbance of sample/Absorbance of sample × 100

### 2.7. Maintenance of Cell Line

The mouse fibroblast (L929) cell line was procured from NCCS, Pune, and was maintained according to the standard protocols. The cells were maintained in Dulbecco’s Modified Eagle Medium (DMEM) supplemented with 10% (*v*/*v*) foetal bovine serum (FBS) and 1% antibiotic, antimycotic solution (1000 U/mL penicillin G, 10 mg/mL streptomycin sulphate, 5 mg /mL gentamycin and 25 μg/mL amphotericin B). The cells were incubated in a humidified atmosphere of 5% CO_2_ at 37 °C.

## 3. In Vitro Studies

### 3.1. Cytotoxicity Evaluation of Extract Using MTT Assay

The cytotoxic evaluation of the fruit extract was carried out as per the procedure [13]. In brief, the cells were seeded in 96-well plates (10^5^ cells/well) and incubated for 24 h. After 24 h of incubation, the fruit extract of *Solanum indicum* at various concentrations ranging from (10–500 μg/mL) was added to each well and incubated for 24 h. After 24 h of post-incubation, the medium in each well was discarded and replaced with serum-free medium (100 μL) containing 0.5 mg/mL of MTT. After 4 h, the MTT was discarded, and the formazan product of the MTT reduction was dissolved in 100 μL of DMSO, and the absorbance was recorded at 570 nm using a microplate reader. The % cell viability was calculated using the formula,

% cell viability = A(s)/Absorbance (c) × 100

where A(s) and A(c) are the absorbance of the test and control samples, respectively.

### 3.2. Cellular Antioxidant Assay

The assay was performed to determine the antioxidant activity in the cell line and was carried out according to the method described [14] with slight modification. In brief, L-929 (mouse fibroblast) cells were seeded in a transparent flat-bottom 96-well plate at 10,000 cells per well and incubated for 24 h. After 24 h of incubation, the cells were washed with phosphate-buffered saline (PBS) and incubated for 30 min with 5 µM dichloro dihydrofluorescin diacetate (DCFH-DA). The cells were then washed again with PBS. To assess the cellular antioxidant activity, the cells were incubated for 1 h with different concentrations (10–100 µM) of Solanum indicum extracts in the presence of 200 µM of 2, 2′-Azobis (2-methylpropionamidine) dihydrochloride. The fluorescence intensity was measured immediately after adding 2, 2-azobis (2-methyl propionamidine) dihydrochloride at an interval of 5 min for 1 h using an excitation wavelength of 485 nm and an emission wavelength of 538 nm. EC_50_ was calculated using the logarithmic regression of the dose-response curve after the subtraction of both the blank and sample fluorescence using the following equation,

CAA Units = 100 − (AUC Antioxidant/AUC Control) × 100

### 3.3. α-Amylase Inhibition Assay

The α-amylase inhibition assay was carried out as per the procedure reported by [15]. In total, 500μL of various concentrations (10, 20, 40, 60, 80, 100 μg/mL) of extract and 500 μL of 0.02 M sodium phosphate buffer (pH 6.9 with 0.006 M NaCl) containing α-amylase solution (0.5 mg/mL) were incubated for 10 min at 25 °C. After pre-incubation, 500 μL of 1% starch solution in 0.02 M sodium phosphate buffer was added to each tube. The reaction mixtures were then incubated at 25 °C for 10 min. The reaction was stopped by adding 1 mL of dinitrosalicylic acid colour reagent. The test tubes were then incubated in a boiling water bath for 5 min and cooled to room temperature. The reaction mixture was then diluted after adding 10 mL of distilled water and the absorbance was measured at 540 nm. The % inhibition was calculated using the formula,

% inhibition= [A (control) − A (test)/A (control) × 100].

## 4. Animal Experimentation

### 4.1. Experimental Animals

Male Albino Wistar rats weighing 180–220 g were selected for the antidiabetic study. The animals were kept at an ambient temperature of 25–30 °C and a relative humidity of 45–55% with a 12 h dark and light cycle. The animals were fed with a standard pellet diet (Hindustan lever, Mumbai) and water ad libitum. The study was approved by the Institutional Animal Ethical Committee, Dept of Pharmacology, College of Veterinary and Animal Sciences, Udgir, (Maharashtra) approval no. VCU/CPCSEA/IAEC/2/14 (II).

### 4.2. Preparation of the Test Samples

The extract and standard drug (Glibenclamide) were suspended in 0.5% carboxy methyl cellulose (CMC) in distilled water prior to oral administration to the experimental animals.

### 4.3. Acute Toxicity Study

The acute oral toxicity study was carried out in accordance with the Organisation for Economic Co-operation and Development (OECD) guideline No.423. The overnight-fasted animals were orally administered with fruit extract at a dose range of (100–2000 mg/kg) to different groups of rats comprising six in each. The animals were observed continuously for one hour for any gross behavioural and neurological changes like drowsiness, restlessness, writhing, convulsions, and symptoms of toxicity and mortality, if any, periodically for the next 6 h, and then again at 24 h for signs of acute toxicity.

### 4.4. Evaluation of Extract in Oral Glucose Tolerance Test

To study the normoglycemic effect of the extract, an oral glucose tolerance test was carried out on the overnight-fasted animals. The animals were divided into four groups of six each.

Group I: Normal control rats treated with vehicle only (0.5% CMC, p.o)

Group II: Rats treated with Glibenclamide (5 mg/kg, p.o)

Group III: Rats treated with *Solanum indicum* extract (100 mg/kg, p.o)

Group IV: Rats treated with *Solanum indicum* extract (200 mg/kg, p.o)

Glucose at 2 g/kg was administered orally 30 min prior to the administration of the extract and glibenclamide [16]. Blood was drawn from the tail vein at regular intervals of 0, 30, 60, 90 and 120 min, and the glucose level was measured using a glucose estimation kit (Merck India Ltd. Mumbai, India).

### 4.5. Induction of Diabetes Mellitus

Experimental diabetes was induced [17] by a single intraperitoneal injection of STZ (60 mg/kg body weight; dissolved in 0.1 M ice-cold citrate buffer (pH 4.5)) in the overnight-fasted animals. Hyperglycemia was confirmed by the elevated blood glucose level and was estimated on day 0 and 72 h after STZ injection. The rats with blood glucose levels of more than 200 mg/dL were used for the study [18].

### 4.6. Experimental Design

The animals were divided into five groups (n = 6) and were treated daily as follows

Group I: Normal control (receives 0.5% CMC)

Group II: Diabetic control

Group III: Diabetic animals treated with glibenclamide (5 mg/kg, p.o)

Group IV: Diabetic animals treated with *Solanum indicum* extract (100 mg/kg, p.o)

Group V: Diabetic animals treated with *Solanum indicum* extract (200 mg/kg, p.o)

The study was carried out for a period of 30 days, and the hypoglycemic potential of the extract was evaluated by estimating the serum glucose levels at regular intervals of 10, 20 and 30 days. Other parameters like serum cholesterol, triglycerides, ALT and AST were also estimated.

### 4.7. Biochemical Assays

Serum total cholesterol (TC), triglycerides (TG) and high-density lipoprotein cholesterol (HDLc) were estimated using the standard protocol and as per the procedures mentioned in commercial kits (Merck Laboratories, Maharashtra, India).

#### 4.7.1. Oxidative Stress Biomarkers

##### SOD

The activity of SOD was measured as per the procedure [19]. In brief, 200 μL of tissue homogenate was added to 2500 μL of 0.05 M carbonate buffer. The reaction was initiated by the addition of 0.3 M adrenaline, and the absorbance was measured at 480 nm for 150 s at an interval of 30 s. Inhibition (50%) in the rate of the auto-oxidation of pyrogallol was measured at 420 nm and expressed as one unit of enzyme activity.

##### CAT

The activity of CAT was measured according to the procedure [20]. In brief, 50 μL of tissue homogenate was added to 1 mL of H_2_O_2_ (30 mM) and 2 mL of phosphate buffer (pH 7), and the absorbance was measured at 240 nm. The molar extinction coefficient of H_2_O_2_ (43.6 M/cm) was used for measuring the activity of the catalase.

##### GPx

The activity of GPx was measured according to the procedure mentioned in the commercial kit (Merck Laboratories, India).

## 5. Statistical Analysis

All the values of the experimental results were expressed as Mean ± standard error of mean (SEM). For the evaluation of the oral glucose tolerance test, a two-way ANOVA followed by a Bonferroni post-test were performed.

Various parameters involved in the antidiabetic study were analysed statistically by applying a one-way analysis of variance (ANOVA) followed by Dunnett’s multiple comparison tests using graph pad prism (version 5.5) computer software. The results were considered statistically significant if *p* < 0.05.

## 6. Result

### 6.1. Preliminary Phytochemical Analysis

Preliminary phytochemical analysis of the extract revealed the presence of alkaloids, glycosides, saponins and phenolic compounds.

### 6.2. Total Phenolic Content

**Figure 1:**
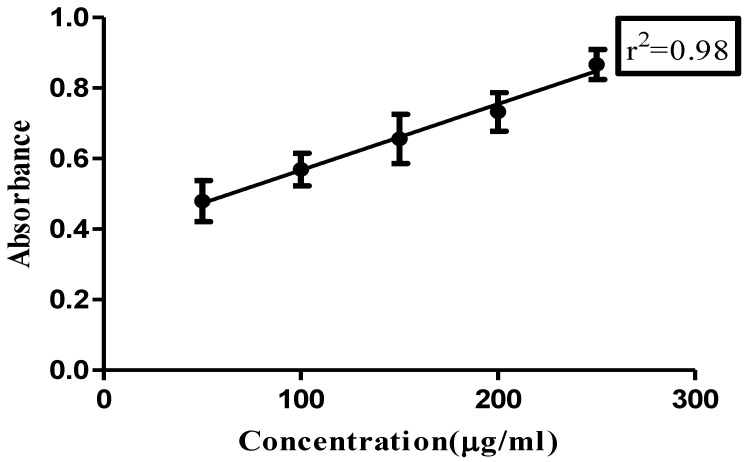
Calibration curve for gallic acid.

The total phenolic content of the extract was found to be 98.5 mg gallic acid equivalent/gram of extract calculated from the gallic acid calibration curve depicted in Figure 1.

### 6.3. DPPH Radical Scavenging Assay

The antioxidant activity of the extract was measured on the basis of the scavenging activity of the stable DPPH radical depicted in Table 1. The extract showed the maximum antioxidant activity of 85.89% at a concentration of 100 µg/mL, and the IC_50_ value was found to be 2.32 µg/mL.

### 6.4. MTT Assay

**Figure 2:**
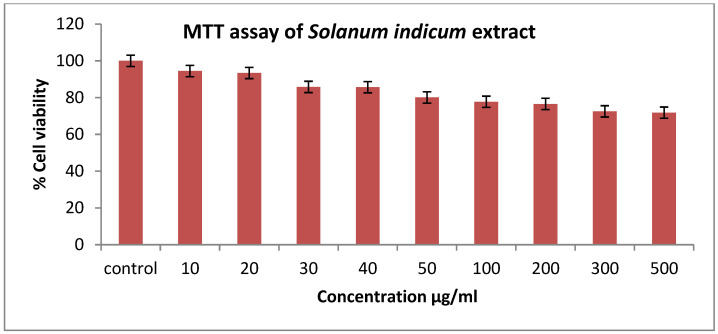
MTT assay of *Solanum indicum* extract.

Treated mouse fibroblast cells (L929 cells) with different concentrations of extract for 24 h did not cause a significant loss in cell viability compared to the untreated cells. The cells treated with a higher concentration (500 µg/mL) of the extract showed 73% cell viability, depicted in Figure 2.

**Figure 3:**
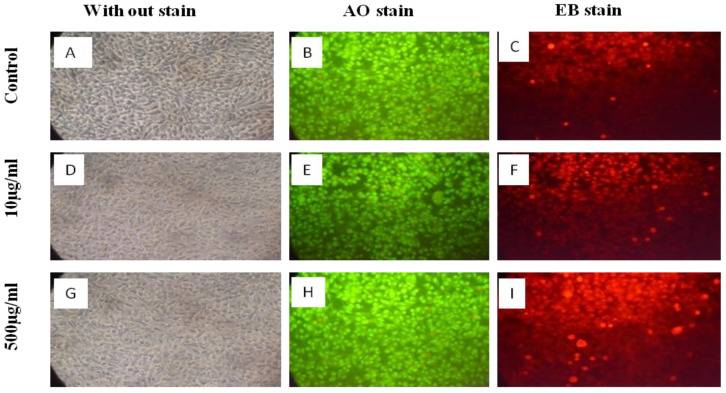
Morphology analyses of L929 cells treated with *Solanum indicum* extract.

Cells treated with the lowest (10 µg/mL) and highest (500 µg/mL) concentrations of the extract did not show altered morphology and retained their viability, confirmed by acridine orange (AO) and ethidium bromide (EtBr) staining, depicted in Figure 3.

Without staining, there was no significant alteration in the cell morphology of the untreated cells and the cells treated with 10 and 500 µg/mL of extract (Figure 3A,D,G); untreated L929 cells emitted intense green fluorescence and weak signals for red fluorescence (Figure 3B,C), L929 cells treated with 10 µg/mL of extract (Figure 3E,F) and L929 cells treated with 100 µg/mL of extract (Figure 3H,I).

### 6.5. Cellular Antioxidant Assay

**Figure 4:**
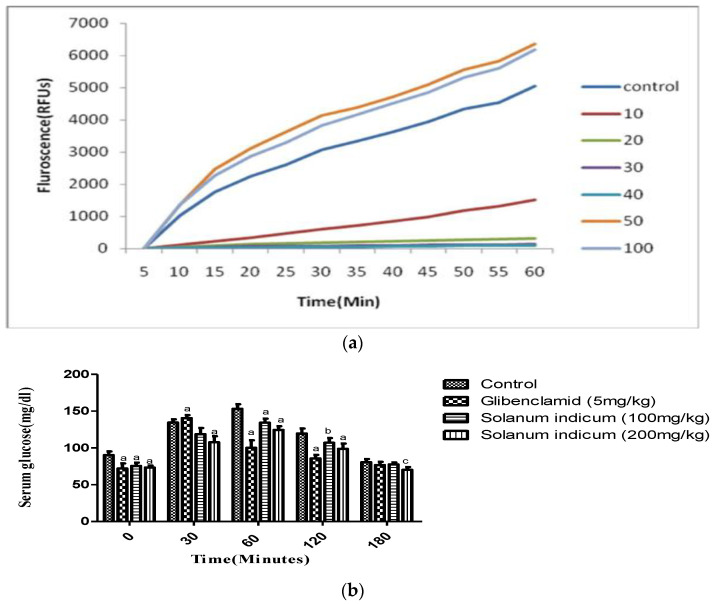
(**a**) Cellular antioxidant activity of the methanolic extract of *Solanum indicum* fruits; (**b**) Oral glucose tolerance test of the extract of *Solanum indicum* fruits. All the values were expressed in Mean ± SEM, where, a (*p* < 0.001), b (*p* < 0.01) and c (*p* < 0.05) when compared with the vehicle control animals.

The cellular antioxidant activity of the extract was evaluated, and the results were expressed in a microgram of quercetin equivalents, depicted in Figure 4a. The EC_50_ of the extract was found to be 14.43 μg/mL.

### 6.6. α-Amylase Inhibition

The inhibitory effect of the extract against porcine pancreatic amylase was shown in Table 2. The fruit extract showed concentration (10, 20, 40, 60, 80 and 100 μg/mL)-dependant α-amylase inhibition. At 100 μg/mL, the inhibitory activity was 83.60%, whereas 10, 20, 40, 60 and 80 μg/mL of the extract showed 38.11, 53.8, 59.66, 66.59 and 72.10% inhibitory activity, respectively. The IC_50_ values of the extract and Acarbose were found to be 17.22 μg/mL and 2.65 μg/mL, respectively.

### 6.7. Acute Toxicity Study

In the acute toxicity study, the oral administration of different doses of extract ranging from (100–2000 mg/kg) does not produce any toxic effects on general appearance, motor function, or behaviour. It revealed the non-toxic nature of the extract. The body weight and food consumption of all animals were normal as compared to the vehicle-treated groups. For further studies, the doses were fixed as 100 and 200 mg/kg.

### 6.8. Effect of Extract on Oral Glucose Tolerance Test

Figure 4b shows the oral glucose tolerance test for the fruit extract. It showed a significant (*p* < 0.001) reduction in blood glucose levels at 200 mg/kg as compared to 100 mg/kg from 30 min onwards and sustained it for 180 min.

### 6.9. Effect on Fasting Blood Glucose Levels

The effect of the extract on fasting blood glucose was depicted in Table 3. A significant reduction was observed in rats treated with the crude extract. The administration of the extract to diabetic rats showed a significant reduction in blood glucose from the 10th day onward (Table 4). The diabetic rats treated with the extract showed a significant (*p* < 0.001) increase in total body weight as compared to the control animals, and the efficiency of the extract was found to be retained in the animal body until the end of treatment, as shown in (Table 5).

STZ-induced diabetic rats treated with two different doses of extract (100 and 200 mg/kg) showed a significant (*p* < 0.01) reduction in blood glucose (Table 5). The extract showed a prominent reduction of 55.93% in the blood glucose at a dose of 200 mg/kg when compared with diabetic rats at the end of 30 days of treatment.

### 6.10. Effect on ALT and AST

The oral administration of SIE for 30 days showed a significant (*p* < 0.01) decrease in the level of AST. SIE at a dose of 200 mg/kg showed a more prominent and significant (*p* < 0.001) effect on reducing the level of ALT compared to the diabetic control animals (Table 6).

### 6.11. Effect on Serum Lipid Profile

An increase in the level of serum lipids like TG and TC and with a decrease in the level of HDLc was observed in STZ-induced diabetic rats. The oral administration of *Solanum indicum* extract reversed the elevated levels of these lipids with a significant (*p* < 0.01) rise in high-density cholesterol (Table 7) in a dose-dependent manner when compared with diabetic rats.

### 6.12. Oxidative Stress Markers

The oral administration of *Solanum indicum* extract for 30 days significantly (*p* < 0.05) decreased the activities of the antioxidant enzymes SOD, CAT and GPx (Table 8) in the liver of STZ-induced diabetic rats.

## 7. Discussion

Diabetes mellitus is a heterogeneous metabolic disorder affecting the major population worldwide and is characterised by hyperglycaemia. The hyperglycaemia associated with diabetes affects various biochemical and cellular metabolic reactions affecting the function of various vital organs. A sustained reduction in blood glucose will decrease the risk of developing microvascular complications [21].

Antioxidants have already been found in herbs and herbal products due to their natural origin. The antioxidants obtained from plants are devoid of various adverse effects compared to synthetic antioxidants [22,23]. The antioxidant activity of the extract was measured on the basis of the scavenging activity of the 1-diphenyl 2-picrylhyorazyl (DPPH) free radical (Table 1). The extract was able to scavenge the stable DPPH radical in a dose-dependent manner with the highest activity at 100 µg/mL (Table 3), depicting the presence of reducing phytochemicals in the extract which reduced the DPPH free radicals, which could be possible mechanisms for the reduction in oxidative stress and the generation of free radicals in diabetes.

Antioxidants present in the crude extract may operate via different mechanisms. The best measure is from human studies or animal models, but these methods are costlier and time-consuming [24]. Hence, we tested the cell-based antioxidant activity of the crude extract for potential bio-activity. This assay revealed that the crude extract played an important role in giving protection against peroxyl radicals under standard physiological conditions, exhibiting its antioxidant potential. An MTT assay was carried out using mouse fibroblast cell lines and showed maximum cell viability at 500 µg/mL, revealing the non-toxic and biocompatible nature of the crude extract. An acute (single dose) toxicity study was carried out in experimental animals to establish the safe dose range as well as to evaluate the long-term toxic effects of the chemicals present in the crude extract. The body weight and food consumption of all animals were normal as compared to the vehicle-treated groups. The oral administration of the extract in doses ranging from (100–2000 mg/kg) does not produce any toxic effects on the motor functions, general appearance and behaviour of rats in acute toxicity studies.

Streptozotocin is commonly used for the induction of diabetes (type I and II) based on the dose employed in experimental animals, which causes the selective destruction of pancreatic β cells via the production of NO. This results in the rapid reduction in the pyridine nucleotide concentration of pancreatic cells followed by the necrosis of β cells. STZ causes various diabetic complications via the generation of superoxide dismutase anions which act on the mitochondria [25,26,27]. Taking this into consideration, the present study was carried out to evaluate the antidiabetic and antioxidant activity of the extract in STZ-induced diabetic rats. To compare the efficacy of various antidiabetic agents in STZ-induced diabetic rats, Glibenclamide was used as a standard drug [28].

The methanolic extract showed dose-dependent hypoglycaemic action; from an oral glucose tolerance test, it was concluded that 200 mg/kg showed the maximum hypoglycaemic effect and sustained it for 3 h. In our study, the extract caused a significant reduction in blood glucose compared with STZ-diabetic rats. This could be due to the stimulation and release of insulin from regenerated pancreatic β cells by the action of inhibiting the ATP-sensitive K^+^ channels like the standard drug Glibenclamide, as well as due to the presence of phenolics and antioxidant compounds in the extract. Previous reports showed that phenolic compounds acted on ATP-sensitive K^+^ channels and controlled hyperglycemia [29], so the phenolic compounds present in the extract may be responsible for insulin-mimetic action.

The extract showed concentration-dependent α-amylase inhibition. These findings state that the extract may be useful in controlling post-prandial hyperglycemia by inhibiting the activity of the enzyme α-amylase, which plays a key role in the digestion of complex sugars to simple sugars which can be absorbed across GIT. Recently, some reports suggested that natural polyphenols could effectively inhibit the activity of carbohydrate hydrolysing enzymes such as α-amylase and α-glucosidase [30]. Therefore, the high contents of phenolic compounds present in the extract may be responsible for controlling post-prandial hyperglycemia. The extract showed a more prominent action on the digestion of starches compared with disaccharide sucrose.

A severe loss in body weight is one of the most important characteristics of STZ-induced diabetes [31], mainly due to increased protein wasting and the non-availability of glucose for the utilisation of energy [32,33]. Diabetic rats treated with the extract increased in body weight. The increase in body weight might be due to improvement in glycaemic control followed by the increased synthesis of structural proteins [34]. However, it remains lesser as compared to normal control animals.

Tissue damage in diabetes mellitus is considered to be mediated by the action of free radicals by attacking the membrane through lipid peroxidation [35], which finally leads to membrane damage and dysfunction [36]. Decreased lipid peroxidation and improvement in the antioxidant status may be one of the mechanisms which may contribute to the prevention of diabetic complications. In our investigation, the methanolic fruit extract of the plant significantly decreased lipid peroxidation which could be due to the antioxidant effects of phenolic and flavonoid compounds which are mainly found in the crude extract and estimated during preliminary phytochemical analysis.

The hyperglycemia associated with diabetes causes the production of free radicals either due to glucose degradation, the non-enzymatic glycation of proteins or subsequent oxidative degradation [37]. Several reports suggested that oxygen-derived free radicals were produced during the process of glycation [38], and these generated free radicals cause lipid peroxidation and damage to cell membranes in diabetes [37]. The changes in lipid peroxidation associated with diabetic animals showed a decrease in the activity of several antioxidant enzymes viz. superoxide dismutase, catalases and glutathione peroxidase, which play a key role in scavenging the toxic intermediates produced during the process of incomplete oxidation. This leads to the excess availability of superoxide anions (O_2_^−^) and hydrogen peroxide in the biological systems due to the decreased activity of these enzymes, which in turn generates hydroxyl radicals responsible for the initiation and propagation of lipid peroxidation [39]. The treatment of diabetic rats with the extract increased the activity of these enzymes and may help in scavenging the generated free radicals.

Diabetes is a metabolic syndrome that affects the majority of critical organs, including the liver [40]. Non-alcoholic fatty liver ailments, including steatohepatitis, cirrhosis and even hepatocellular carcinomas, could be raised by the interruptions of lipid, carbohydrate and protein metabolisms associated with diabetes. High glucose-provoked augmented oxidative stress plays a pivotal role in hyperglycemia-endorsed liver diseases [41]. Thus, to explore the effect of our test material on diabetes-manifested hepatic injury, we tested the biochemical parameters of sera associated with liver damage as well as the redox status of the liver upon the treatment with the extract. ALT and AST are the marker enzymes for liver function. Necrosis caused by STZ results in elevated levels of serum ALT and AST due to leakage from the liver cytosol [42] Increased levels of these enzymes indicate potential liver damage and hepatotoxicity [42,43]. The treatment of diabetic rats with the extract at two different doses significantly reduced the activity of these enzymes compared to diabetic animals, revealing the hepatoprotective nature of the extract.

The development of microvascular and macrovascular complications like atherosclerosis and coronary artery diseases are attributed to elevated levels of serum cholesterol and triglycerides [44]. The treatment of diabetic rats with the extract for 30 days significantly reduced serum triglycerides and total cholesterol and was able to modulate blood lipid abnormalities.

## 8. Conclusions

In the present study, the extract showed a beneficial effect on the blood glucose level. It also restored the altered serum triglycerides, cholesterol and enzymes like AST and ALT. It modulated and enhanced the level of endogenous antioxidants such as catalase, superoxide dismutase and glutathione peroxidase. The results of the study showed that *Solanum indicum* fruit extract possesses antidiabetic and antioxidant activity, and it was comparable to Glibenclamide. The results of the study may possibly be used for the development of new antidiabetic drugs for the management and treatment of type II diabetes. Further research is required for identifying the molecular targets and isolation of bioactive phytoconstituents to find out the molecular mechanism for its antidiabetic action.

## Figures and Tables

**Table 1:** DPPH scavenging assay of SIE.

Concentration of (μg/mL)	DPPH Scavenging Activity (%)
10	21.29
20	46.09
30	72.87
40	79.86
50	82.06
100	85.89

**Table 2:** α-Amylase inhibition assay.

Treatments	Concentration (µg/mL)	% Inhibition	IC50 (µg/mL)
Fruit extract of *Solanum indicum*	10	38.11 ± 0.37	17.22
	20	53.80 ± 0.31	
	40	59.66 ± 0.64	
	60	66.59 ± 0.52	
	80	72.10 ± 0.07	
	100	83.60 ± 0.09	
Standard (Acarbose)	10	55.92 ± 0.31	2.65
	20	65.73 ± 0.56	
	40	73.83 ± 0.73	
	60	82.55 ± 0.77	
	80	82.86 ± 0.49	
	100	93.61 ± 0.56	

**Table 3:** Effect of SIE on the fasting blood glucose levels of STZ-induced diabetic rats.

Group	Treatment	0 h	1 h	2 h	3 h	% Reduction
I	Diabetic control	387.2 ± 5.1	396.5 ± 4.3	398.8 ± 3.8	394.6 ± 3.8	-
II	Glibenclamide (5 mg/kg)	344.3 ± 1.0	174.7 ± 1.2	115.9 ± 2.2	106.6 ± 2.7	69.03
III	*Solanum indicum* extract (100 mg/kg)	389.7 ± 4.8	347.6 ± 3.6	316.5 ± 3.9	286.4 ± 6.2	26.50
IV	*Solanum indicum* extract (200 mg/kg)	355.2 ± 4.7	323.6 ± 3.4	279.1 ± 5.6	244.4 ± 4.3	31.19

**Table 4:** Effect of the extract of *Solanum indicum* fruits on serum glucose in STZ-induced diabetic rats.

Group	Treatments	0 Days	10 Days	20 Days	30 Days
I	Control (0.3% *w*/*v* CMC)	95.8 ± 1.88	93.2 ± 0.86	93.200 ± 0.86	89.800 ± 0.8
II	Diabetic control	287.4 ± 1.88	281.8 ± 2.03	275.8 ± 1.39	272.8 ± 1.98
III	Glibenclamide (5 mg/kg)	179.4 ± 1.6	133.8 ± 2.27 **	125.8 ± 3.29 **	122.8 ± 1.93 **
IV	*Solanum indicum* extract (100 mg/kg)	180.8 ± 1.39	138.2 ± 2.55 *	132.2 ± 2.85 *	122.8 ± 1.93 *
V	*Solanum indicum* extract (200 mg/kg)	180.2 ± 1.24	136 ± 2.72 *	125.4 ± 2.06 *	120.2 ± 1.65 *

All values were expressed in Mean ± SEM of six animals. ** *p* < 0.0001, as compared with the control group * *p* < 0.01, as compared with the diabetic control group.

**Table 5:** Effect of SIE on body weight in STZ induced diabetic rats.

Group	Treatments	Initial	Final
I	Control (0.3% *w*/*v* CMC)	198.8 ± 3.26	200.0 ± 1.41
II	Diabetic control	161.4.4 ± 1.2	141 ± 1.0
III	Glibenclamide (5 mg/kg)	162.4 ± 1.7	181.1 ± 1.50 **
IV	*Solanum indicum* extract (100 mg/kg)	163.2 ± 1.7	175.6 ± 1.56 **
V	*Solanum indicum* extract (200 mg/kg)	163.6 ± 2.15	178.4 ± 1.63 **

All the values were expressed in Mean ± SEM of six animals. ** *p* < 0.001, as compared with the control group.

**Table 6:** Effect of SIE on ALT and AST levels in STZ-induced diabetic rats.

Group	Treatment	AST	ALT
I	Control	36.43 ± 2.48	54.68 ± 5.48
II	Diabetic control	66.46 ± 4.25	90.88 ± 3.52
III	Glibenclamide (5 mg/kg)	42.31 ± 4.89 ***	53.67 ± 3.65 ***
IV	*Solanum indicum* (100 mg/kg)	48.36 ± 3.41 **	72.36 ± 5.41 **
V	*Solanum indicum* (200 mg/kg)	45.89 ± 3.21 **	61.36 ± 5.63 ***

All the values were expressed in Mean ± SEM of six animals. *** *p* < 0.001, as compared with the control group. ** *p* < 0.01, as compared with the diabetic control group.

**Table 7:** Effect of the extract of *Solanum indicum* fruits on lipid profile in STZ-induced diabetic rats.

Treatment	TC	TG	HDLc
Control	166.7 ± 5.1	140 ± 2.6	43 ± 1.3
Diabetic control	213.8 ± 3.6 **	182 ± 3.8 **	25 ± 2.8 *
Glibenclamide	153.6 ± 4.3 *	135 ± 4.2	40 ± 3.1
*Solanum indicum* 100 mg/kg	186.8 ± 3.9	175 ± 2.8 *	26 ± 4.3
*Solanum indicum* 200 mg/kg	168.2 ± 5.3 *	160 ± 4.1 *	32 ± 2.9 *

All the values were expressed in Mean ± SEM of six animals ** *p* < 0.001, as compared with the control group, * *p* < 0.01 as compared with the control group.

**Table 8:** Changes in SOD, CAT and GPx activities of normal and diabetic rats after 30 days of treatment with the extract of *Solanum indicum* fruits.

Treatments	SOD (U/mg of Protein)	CAT (mM of H_2_O_2_ Decomposed/min)	GPx (U/mg of Protein)
Control	4.89 ± 0.06	59.78 ± 2.86	44.35 ± 1.69
Diabetic control	2.70 ± 0.08	36.43 ± 3.10	26.72 ± 2.10
Glibenclamide (5 mg/kg)	4.58 ± 0.02 *	58.93 ± 1.68 *	35.31 ± 2.22 *
*Solanum indicum* (100 mg/kg)	3.76 ± 0.09 *	41.65 ± 1.56 *	37.49 ± 1.65 *
*Solanum indicum* (200 mg/kg)	4.32 ± 0.03 **	56.53 ± 2.33 *	40.52 ± 2.76 *

* *p* < 0.01 when compared to the day 1 value of the same group ** *p* < 0.001 when compared to the day 1 value of the same group.

## Data Availability

As mentioned in table.
